# Dental Education

**Published:** 1883-05

**Authors:** J. A. Watling


### Dental Education.

BY J. A. WATLING, D. D. S.

Read before the Michigan Dental Society.

MR. PRESIDENT AND GENTLEMEN :

The subject of Dental Education, which has been assigned to me, has been so thoroughly written upon and discussed, that there don't seem to be anything left to be advanced regarding it. It is one that has received much attention from the profession of late, and presents a field that is open wide for discussion. It is one into which the profession may well throw its influence, for many changes for the better may be instituted. In glancing over our many journals for some ideas upon this subject, I find almost as many theories as there are speakers, ideas conflicting and ideas commingling; I shall only try to give you a few hasty thoughts to open the discussion.

To start out, all would seem to acknowledge the importance of a good primary education. If so, and I think none will dissent, why not all of our dental schools make *this* the first requisite to enter their portals? This would be the *first step* in the *advance* of Dental Education. As to the necessity or propriety of a Collegiate Education there is room for discussion of wider range than I shall take in this paper. But this important fact we must keep in mind—that the average human brain is only capable of grasping and retaining a given amount of knowledge, of course some more than others; now accepting this as a fact, is there not danger that the manipulative ability, which must be paramount, may be crowded out at the expense of the higher education? These are mere thoughts present for your discussion.

It may also be claimed that the brain can receive knowledge at but a given rate of speed; admitting this to be a fact, I would suggest, that the student is *not* given *sufficient time* in our dental schools to grasp and digest the subjects taught; it is too much of a confused jumble; neither are the classes *graded* to the best advantage. The student should not be expected to take all of the subjects of the curriculum in a single year, but should be allowed to pursue such studies, as properly belong to each class-year. I would suggest that the terms be lengthened to a full college year of nine months, and that none should be graduated in less than two full terms. I have found by experience that the time spent in drilling students in the manipulative art is far too short. I am aware that some will say, that it is injustice to keep *all* the same length of time, that some will learn as much in six months as others will in twelve. I will admit that. It is also claimed the quick are held back, waiting for the dull to learn; but that is not the fact in the case; there is no holding back; there is no obstacle placed in the way of the bright and quick student; on the contrary he is allowed to make all the progress he can, and *I have never seen one too well qualified*, or one who had lost any time by staying two full terms; on the contrary, those who have been best qualified have frequently expressed regret at not being able to stay longer; of course all cannot be equally well qualified, no length of time could make them so.

Another thought I may suggest for your discussion: Is it an advantage or a disadvantage to the student to have a great *multiplicity of teachers* in the clinical department? So far as my experience goes, in that way, it has been a disadvantage; for what reason? Because no two of these instructors use the same instruments or operate alike, but each has a method peculiar to himself, which, like all the rest of us, he thinks is best, and the only one to be adopted; and he must impress this very strongly on the mind of the student, or he will *not* have made as favorable an impression as his *predecessor* did the week before. Thus the student receives these new methods and ideas once a week, before he has any well grounded principles of his own; and thus from week to week he is trying new methods, and not settling down to any one well chosen principle of operating. To illustrate: I recently met one of these instructors, and he told me of his last exploit in that line. He is what we would call one of those extreme operators, and is quite an inventor, having a rubber dam, rubber dam clamp, and pluggers all of his own invention, while his peculiar way of operating is very peculiar to himself, and the only right way, of course. Well, he went to the college where he is announced as an instructor and held a clinic for a half day. Now, what do you think that class did? Why, they called a meeting and passed resolutions immediately, adopting his method of operating, together with his rubber dam clamp and pluggers. Thus you see the instructions of the regular teacher, who, by the way, is a very fine operator and a gentleman, are set aside in a half day's work. I am unable to say, if the class did this every week or not, as I have not seen any other member of the corps of teachers.

I am very firmly of the opinion, that only one system of instruction should be taught at a time. After a student has become a competent operator, he can then learn much by seeing *others* operate, but not while he is getting the first principles—it is feeding the brain too fast.

It has been said that the dentist, like the artist, must be *born.* There is much in this, no matter how much education, science and theory a man may have, if he has not the natural manipulative ability, he cannot be a competent *dentist.* He may go to dental associations, *write well, talk well,* he may be even "*medically educated,*" but if he is a poor *operator*, he is a curse to his patients, a dead load on the profession.

I don't wish to be understood, as disparaging a thorough medical education, or the degree of M.D., though I don't consider the latter an *essential.* It is the *knowledge* that makes the dentist, and not *degree*—and, I think, none will dispute, but that the knowledge can be acquired in a properly conducted dental college. From experience I am prepared to say that the degree of M.D. does not prepare a student to graduate in a dental college in *one term;* while it has often been done, those students do not make their mark with the student that has had his three years' drill in the dental college, not even in years after. It is not to the man who is constantly holding up his M.D. that we look for the most skillful operations, as more than 90 per cent of our operations are of a manipulative character. I may say that the one who serves his patients best is the one whose *fingers* are the best educated. That a high degree of mechanical training is important, none can doubt. We find in our profession many useful men who have come from the various mechanical trades—the jewelers, machinists, blacksmiths and even tailors have contributed in making fine operators.

In glancing over the journals one is struck with this often repeated expression "medically educated dentists!" I suppose I understand what is meant to be conveyed by the phrase; but think they might express their meaning a little clearer, if they would say, "having the degree of M.D., as all *good* dentists are educated *medically*, consequently *medically educated.* If they wish the distinction let them make it plain and just. Now let us look and see where all this breeze comes from about "medically educated" dentists! Does it come from the best operators? or from the profession at large? or from the *medical profession?* Does it come from eminent dentists who have spent three long years in some medical school earning the degree of M.D., or finally does it come from those who have spent a short time in some short-term college, or through the influence of friends and cheek, have bulldozed some college to confer the degree of M.D. upon them, and thus all at once, they are suddenly raised to the dignity of being "medically educated?"

Gentlemen, I have just given you these thoughts for discussion, I have set up the pins, please knock them down.

